# Identification and characterization of AckA-dependent protein acetylation in *Neisseria gonorrhoeae*

**DOI:** 10.1371/journal.pone.0179621

**Published:** 2017-06-27

**Authors:** Deborah M. B. Post, Birgit Schilling, Lorri M. Reinders, Alexandria K. D’Souza, Margaret R. Ketterer, Steven J. Kiel, Aroon T. Chande, Michael A. Apicella, Bradford W. Gibson

**Affiliations:** 1 Buck Institute for Research on Aging, Novato, California, United States of America; 2 Department of Microbiology, The University of Iowa, Iowa City, Iowa, United States of America; 3 Department of Pharmaceutical Chemistry, University of California, San Francisco, California, United States of America; University of Florida, UNITED STATES

## Abstract

*Neisseria gonorrhoeae*, the causative agent of gonorrhea, has a number of factors known to contribute to pathogenesis; however, a full understanding of these processes and their regulation has proven to be elusive. Post-translational modifications (PTMs) of bacterial proteins are now recognized as one mechanism of protein regulation. In the present study, Western blot analyses, with an anti-acetyl-lysine antibody, indicated that a large number of gonococcal proteins are post-translationally modified. Previous work has shown that N^ε^-lysine acetylation can occur non-enzymatically with acetyl-phosphate (AcP) as the acetyl donor. In the current study, an acetate kinase mutant (1291*ackA*), which accumulates AcP, was generated in *N*. *gonorrhoeae*. Broth cultures of *N*. *gonorrhoeae* 1291wt and 1291*ackA* were grown, proteins extracted and digested, and peptides containing acetylated-lysines (K-acetyl) were affinity-enriched from both strains. Mass spectrometric analyses of these samples identified a total of 2686 unique acetylation sites. Label-free relative quantitation of the K-acetyl peptides derived from the *ackA* and wild-type (wt) strains demonstrated that 109 acetylation sites had an *ackA*/wt ratio>2 and p-values <0.05 in at least 2/3 of the biological replicates and were designated as “AckA-dependent”. Regulated K-acetyl sites were found in ribosomal proteins, central metabolism proteins, iron acquisition and regulation proteins, pilus assembly and regulation proteins, and a two-component response regulator. Since AckA is part of a metabolic pathway, comparative growth studies of the *ackA* mutant and wt strains were performed. The mutant showed a growth defect under aerobic conditions, an inability to grow anaerobically, and a defect in biofilm maturation. In conclusion, the current study identified AckA-dependent acetylation sites in *N*. *gonorrhoeae* and determined that these sites are found in a diverse group of proteins. This work lays the foundation for future studies focusing on specific acetylation sites that may have relevance in gonococcal pathogenesis and metabolism.

## Introduction

*Neisseria gonorrhoeae*, the etiologic agent of gonorrhea, is a growing public health concern. The World Health Organization (WHO) recently estimated that 78 million new cases of gonorrhea occur annually [1]. In the United States, gonorrhea is the second most commonly reported notifiable disease [2]. *N*. *gonorrhoeae* infection can occur in both men and women, but the mechanisms and development of the infections differ. Infection in men is typically symptomatic, causing localized inflammation of the urethra with urethral discharge, so treatment is sought [3, 4]. In women, 40–80% of those infected are asymptomatic and therefore they may not seek treatment [3–8]. Untreated gonococcal infections in women can lead to chronic infections, an increased risk for infertility and ectopic pregnancy, and an increased risk for acquiring other sexually transmitted infections such as HIV [2–4, 9, 10]. The increased level of multi-drug resistant *N*. *gonorrhoeae* strains has become a cause for concern. Thus the Centers for Disease Control (CDC) recently recommended a change from antibiotic treatment with oral or injectable cephalosporin to using only injectable therapy as a means to slow the spread of antibiotic resistance to this antibiotic [2, 11, 12]. The increased incidence of infection, combined with the increase in strains with multi-drug resistance, led the CDC to prioritize *N*. *gonorrhoeae* as an “urgent” public health threat that needed to be addressed aggressively [13].

Post-translational modifications (PTMs) play an important role in modifying the functions of proteins. N^ε^-lysine acetylation may affect proteins by modulating DNA binding affinity, protein-protein interactions, enzyme activity, substrate binding, protein stability, and protein localization [14–17]. N^ε^-lysine acetylation has long been recognized as a PTM in eukaryotic systems [15, 16, 18]. Subsequent studies in *Salmonella enterica* showed that this PTM also occurs in bacteria [19, 20]. Since this initial discovery, several proteomic studies, with both Gram-positive and Gram-negative bacteria, have found hundreds of lysine-acetylated proteins [21–35] and were recently reviewed[14, 36, 37]. These proteins were shown to be involved in various cellular processes, including central metabolism, translation, transcription, signal transduction, stress response, and virulence [14, 36]. However, to our knowledge, no large-scale proteomic acetylome studies have been performed for *N*. *gonorrhoeae*.

**Fig 1:**
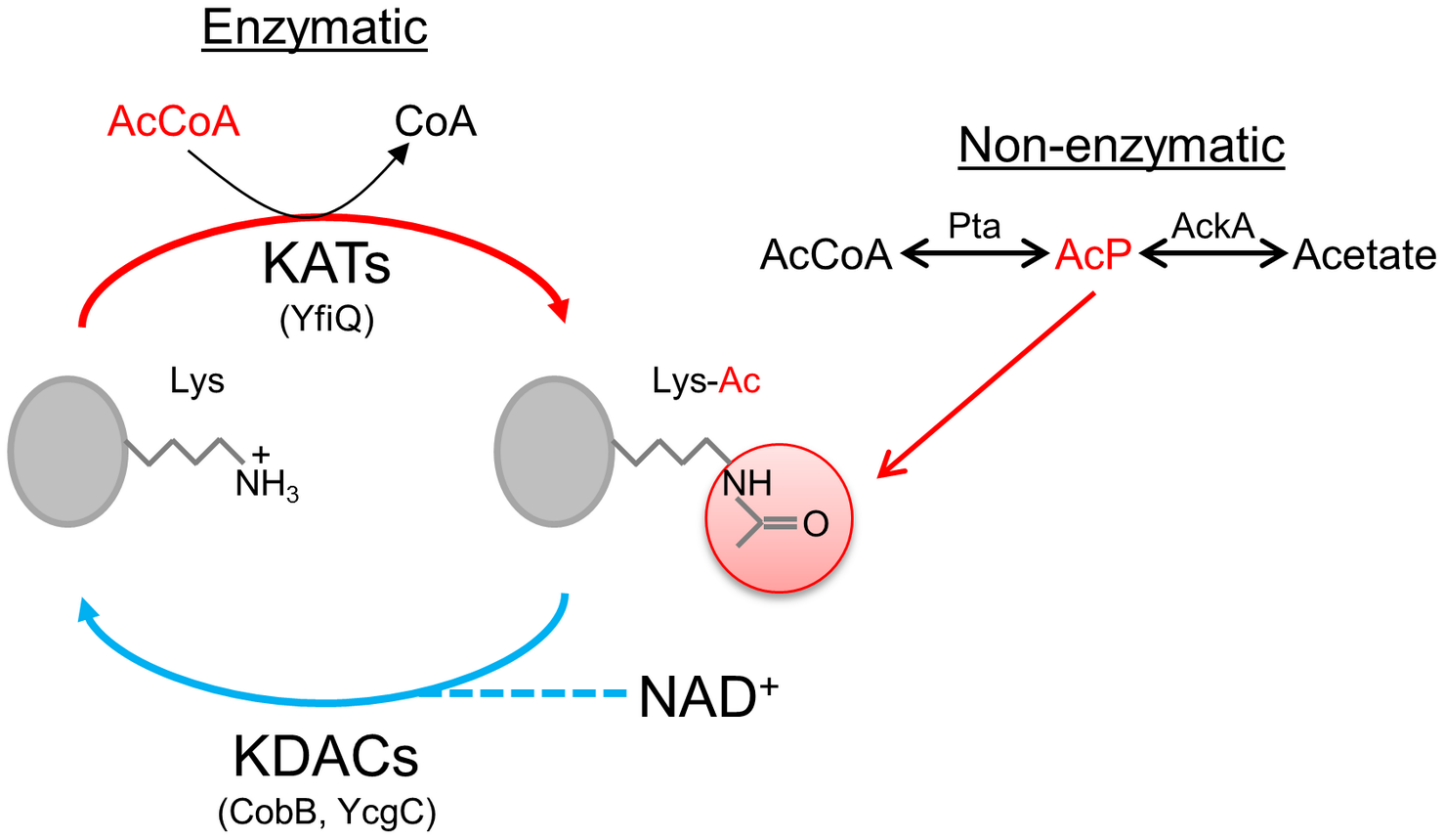
Possible mechanisms of lysine acetylation and deacetylation. Lysine acetyl-transferases (KATs), such as YfiQ, utilize acetyl-CoA (AcCoA) to donate an acetyl group to lysine. Lysine deacetylases (KDACs), such as CobB, can deacetylate some lysines in a NAD^+^-dependent manner. While other KDACs, such as YcgC, deacetylate in a NAD^+^-independent manner. Sequence alignments suggest that KATs and at least one KDAC may be present in *N*. *gonorrhoeae*. Non-enzymatic acetylation utilizes acetyl-phosphate (AcP), the high-energy intermediate in the Pta-AckA pathway, as the acetyl donor.

N^ε^-lysine acetylation has typically been thought to occur enzymatically through the action of lysine acetyltransferases (KATs) transferring an acetyl group from acetyl-coenzyme A (AcCoA) to the ε-amino group of lysine, generating an acetylated-lysine (K-acetyl) (Fig 1). However, recent work in *Escherichia coli* has shown that this process can also occur non-enzymatically, with acetyl-phosphate (AcP) serving as the acetyl group donor (Fig 1) [23, 31]. The level of available AcP seems to impact the level of non-enzymatic acetylation [23, 31]. Levels of AcP are modulated by the phosphotransacetylase (Pta)-acetate kinase (AckA) pathway [38]. In this pathway, AcP serves as the high-energy intermediate, between conversion of AcCoA to acetate [38]; therefore, altering the functions of either Pta or AckA can affect the availability of AcP and can alter the levels of N^ε^-lysine acetylation [23, 31, 38].

In the current study, we wanted to determine if *N*. *gonorrhoeae* proteins are able to undergo non-enzymatic lysine acetylation. Since the availability of AcP is one limiting factor in non-enzymatic acetylation of lysines [23, 31] an acetate kinase mutant (*ackA*), which accumulates AcP [38], was generated. Affinity enrichment of acetylated peptides from broth-grown *N*. *gonorrhoeae* strain 1291 (1291wt) or its isogenic *ackA* mutant (1291*ackA*), followed by high-resolution mass spectrometric analyses, was used to identify acetylation sites. Relative quantitation of the acetylated peptides from the wt versus the *ackA* mutant strain was performed to identify acetylation sites impacted by the *ackA* mutation. Since a metabolic pathway was disrupted in the *ackA* mutant, comparative growth studies under aerobic and anaerobic conditions were performed to test for differences. Additionally, time-course studies of 1291wt and 1291*ackA* biofilms were conducted to compare their formation, maturation, and viability.

## Materials and methods

### Bacterial strains and growth conditions

*N*. *gonorrhoeae* strain 1291, a piliated clinical isolate that has been previously described [39], was used as the parent strain in this study. Bacteria were first grown from frozen stock cultures on GC agar plates (Becton Dickinson, Franklin Lakes, NJ) supplemented with 1% IsoVitaleX (Becton Dickinson) at 37°C with 5% CO_2_. Mutant strains were grown on GC agar plates with 50 μg/mL kanamycin. Subsequently, bacteria for the acetylome experiments were grown in 500 mL cultures of GC broth supplemented with 1% IsoVitaleX and Kellogg’s supplement (see supplemental materials) at 37°C, rotating at 150 rpm, overnight.

To measure growth of 1291wt and 1291*ackA* under aerobic conditions a 24 h growth curve was performed. Plate-grown organisms were used to inoculate IsoVitaleX-supplemented GC broth cultures to OD_600nm_ = 0.1. Duplicate cultures were grown for each strain at 37°C with agitation. Colony forming units (CFUs) were plated in triplicate and counted at 0, 1, 2, 3, 4, 5, 15 and 24 h. The average counts for each time point was then determined.

To measure growth under anaerobic conditions, plate-grown cultures of 1291wt and its corresponding *ackA* mutant were used to inoculate IsoVitaleX-supplemented GC broth cultures and grown to a concentration of 2 x 10^8^ CFU/mL in GC broth. Then 5 μl (1 x 10^6^ CFU) of these cultures were used to inoculate IsoVitaleX-supplemented GC plates containing 1.2 mM sodium nitrite, as previously described [40]. The plates of each strain were then placed in either an anaerobic chamber or grown aerobically at 37°C for 48 h. Plates were grown in triplicate and the experiment was repeated on two separate occasions.

### Biofilm growth in continuous flow chambers

*N*. *gonorrhoeae* 1291wt and 1291*ackA* were grown in continuous-flow chambers using IsoVitaleX supplemented GC broth. Biofilms were formed in a 47 mm circular chamber on a 25 mm glass cover slip, which was sealed using a rubber gasket and screws that fasten the top and bottom portions together. Plate-grown organisms were used to inoculate GC broth cultures to an OD_600nm_ = 0.1. Samples were then grown until the OD_600nm_ = 0.3, and 10 mL of this culture was used to inoculate the biofilm chambers. Chambers were incubated under static conditions at 37°C for 1 h to allow attachment to the glass surface. A flow rate of 60 μL/min was applied to biofilm chambers and allowed to proceed at 37°C for 6, 24, or 48 h. Following incubation, the biofilm effluent was cultured to assess purity, and the chambers were stained with LIVE/DEAD stain (Thermo Fisher Scientific, Waltham, MA) for 30 min followed by treatment with a 4% paraformaldehyde solution for 45 min. Samples were imaged with a Zeiss LSM710 confocal microscope and the stacked images were analyzed using Imaris-8 3D image processing (Oxford Instruments, Abingdon, United Kingdom) for surface-rendered images and statistical analysis (Mann-Whitney comparison) of mass and height of the biofilms. Biofilm experiments were performed in duplicate in two independent experiments.

### Generation and characterization of the *ackA* mutant

The whole *ackA* gene, except for the first and the last codon of the *ackA* ORF, was replaced with a kanamycin resistance cassette. The construction of this mutant by removal of the gene was to ensure that the strain could not revert back to a wild-type strain. Approximately 500 bp upstream and downstream of *ackA* (the “arms of *ackA*”) and the kanamycin resistance cassette were PCR amplified using the primers described in S1 Table. The resulting PCR products were then ligated using PCR SOEing [41]. The final product was purified using IBI Gel/PCR DNA Fragment Extraction Kit (IBI Scientific, Peosta, IA) and then transformed into *N*. *gonorrhoeae* strain 1291. The resulting transformants were screened on GC plates containing 15 μg of ribostamycin/mL. The sequences of mutant transformants (1291*ackA*) were confirmed by PCR and DNA sequencing by the Iowa Institute of Human Genetics, Genomics Division (Iowa City, IA). All PCR reactions were performed using Platinum Pfx DNA polymerase (Life Technologies, Carlsbad, CA) by the “touchdown” PCR method [42]. The constructed 1291*ackA* mutant strain is a nonpolar mutant as both the upstream and downstream genes from *ackA* are divergently transcribed.

### Attempts at complementation of the *ackA* deletion

Complementation of the *ackA* gene was attempted using two methods, plasmid and chromosomal-based complementation. For the plasmid based approach, the *ackA* gene was cloned into the the gonococcal plasmid pLES98 [43]. This construct was then used to transform strain 1291*ackA*. No colonies were obtained on GC agar plates with chloramphenicol selection (5 μg/ml) after multiple attempts using this method. Chromosomal complementation was attempted using the following approach. The full-length *ackA* gene was amplified from strain 1291 with AccI and SmaI restriction sites at the 5’ and 3’ ends, respectively. The product was cloned into pGEMT, followed by ligation into AccI/SmaI-digested pCTS#33. The pCTS#33 construct is a variation of the previously described construct pCTS#32 [44]. In pCTS#33, the plasmid backbone is still pCR2.1 TOPO (Invitrogen) but the *porA* gene from *N*. *gonorrhoeae*, instead of the *porB* gene, flanks the spectinomycin resistance cassette in this construct. The gene for complementation in both constructs is cloned into a multiple-cloning site (MCS) downstream of the spectinomycin resistance cassette, and gene expression is driven by the spectinomycin resistance cassette promoter. The *ackA* complementation construct was transformed into the *N*. *gonorrhoeae ackA* deletion mutant, and transformants were screened for on GC agar supplemented with 50 μg/ml spectinomycin. The sequences of over 20 transformants were analyzed by PCR and sequence analysis. None of the transformants were complements of the *ackA* mutation as all had nucleotide changes that were not present when the *ackA* gene was introduced to the bacteria. Other studies in our laboratory indicated that *ackA* is a tightly regulated gene and we suspect that our attempts at complementation did not work because of the strength and constitutive nature of the spectinomycin promoter used to drive the expression of *ackA*.

### Western blot analyses

Lysates of 1291wt and 1291*ackA* were prepared from broth-grown cultures from 3, 6, and 24 h of growth. Then, equivalent amounts were separated in Novex NuPAGE 4–12% gradient BisTris gels (Thermo Fisher Scientific). Duplicate gels were run and subsequently transferred to nitrocellulose membranes. Membranes were blocked with 4% milk in TBST (“Western Buffer”) and incubated overnight at 4°C with either acetylated-lysine mouse mAb Ac-K-103 (Cell Signaling Technology, Danvers, MA), diluted 1:1000 or the loading control MAb 2C3 [43],diluted 1:100, in 4% milk/TBST. The membranes were washed in TBST and probed with GAM-IgG/HRP conjugate (Jackson ImmunoResearch, West Grove, PA), diluted 1:10,000 in 4% milk/TBST. Following another series of TBST washes, the membranes were incubated with SuperSignal West Pico chemiluminescent substrate (Thermo Fisher Scientific) and developed.

### Sample preparation and proteolytic digestion

Cell pellets from 500 mL cultures of 1291wt or 1291*ackA* were washed twice with PBS and centrifuged at 4°C, 2500 x *g* for 20 min per wash. The cell pellet was re-suspended in HPLC grade water, heated at 65°C for 10 min to kill the bacteria, and lyophilized. The lyophilized samples were re-suspended and denatured in a solution of 6 M urea, 100 mM Tris, 75 mM NaCl containing the deacetylase inhibitors 1 mM tricostatin A and 3 mM nicotinamide. Samples were sonicated on ice (5X each), and cellular debris was removed by centrifugation at 15,000 x *g* for 20 min. Then, protein lysates were reduced with 20 mM dithiothreitol (37°C for 30 min), and subsequently alkylated with 40 mM iodoacetamide (30 min at RT in the dark). Samples were diluted 10-fold with 100 mM Tris pH 8.0 and incubated overnight at 37°C with sequencing grade trypsin (Promega, Madison, WI) added at a 1:50 enzyme:substrate ratio (wt/wt). Samples were acidified with formic acid and desalted using HLB Oasis SPE cartridges (Waters Corp., Milford, MA). Proteolytic peptides were eluted, concentrated to near dryness by vacuum centrifugation, and re-suspended in NET buffer (50 mM Tris-HCl, pH 8.0,100 mM NaCl, 1 mM EDTA). A small aliquot of each protein digestion was further desalted with C-18 ZipTips (Millipore, Billerica, MA), according to standard procedures, for total protein lysate mass spectrometric analysis.

### Affinity purification of lysine-acetylated peptides

The polyclonal anti-acetyl-lysine agarose bound antibody conjugate from ImmuneChem (ICP0380-100) (Burnaby, Canada) was prewashed and incubated with 1 mg digested protein lysate in NET buffer, overnight at 4°C, on a rotating platform. Beads were washed three times in NET buffer, and the enriched acetyl peptides eluted by washing three times with 1% trifluoroacetic acid/40% acetonitrile. Samples were concentrated to near dryness by vacuum centrifugation and resuspended in a 0.1% formic acid/1% acetonitrile solution and de-salted using C-18 ZipTips prior to mass spectrometric analyses.

### Mass spectrometric analyses

All samples were analyzed by reverse-phase HPLC-ESI-MS/MS using an Eksigent UltraPlus nano-LC 2D HPLC system (Dublin, CA) connected to an orthogonal quadrupole time-of-flight (QqTOF) TripleTOF 5600 mass spectrometer (SCIEX, Framingham, MA). Typically, mass resolution for MS1 scans and corresponding precursor ions was ~35,000 while resolution for MS2 scans and resulting fragment ions was ~15,000 (‘high sensitivity’ product ion scan mode). Briefly, after injection, peptide mixtures were loaded onto the analytical C18-nanocapillary HPLC column (C18 Acclaim PepMap100, 75 μm I.D. × 15 cm, 3 μm particle size, 100 Å pore size, Dionex, Sunnyvale, CA) and eluted at a flow rate of 300 nL/min using the following gradient: 5% solvent B in A (from 0–5 min), 5–8% solvent B in A (from 5–12 min), 8–35% solvent B in A (from 12–67 min), 35–80% solvent B in A (from 67–77 min), 80% solvent B in A (from 77–87 min), with a total runtime of 120 min including mobile phase equilibration. Solvents were prepared as follows, mobile phase A: 2% acetonitrile/98% of 0.1% formic acid (v/v) in water, and mobile phase B: 98% acetonitrile/2% of 0.1% formic acid (v/v) in water. Data-dependent acquisitions (DDA): The nanospray needle voltage was typically 2,500 V in HPLC-MS mode. For collision induced dissociation tandem mass spectrometry, the mass window for precursor ion selection of the quadrupole mass analyzer was set to ±1 *m/z*. The precursor ions were fragmented in a collision cell using nitrogen as the collision gas. In DDA mode, the 30 most abundant parent ions were selected for MS/MS analysis following each MS1 survey scan (approx. 50 ms per MS/MS). Dynamic exclusion features were based on value M not *m/z* and were set to an exclusion mass width 50 mDa and an exclusion duration of 30 s. Data-independent acquisitions (DIA), SWATH acquisitions: In the SWATH-MS2 acquisition, a Q1 window of 25 *m/z* was selected to cover the mass range of *m/z* 400–1000 in 24 segments (24×80 ms), yielding a cycle time of 2.25 s, which includes one 250 ms MS1 scan. SWATH-MS2 produces complex MS/MS spectra, which are a composite of all the analytes within each selected Q1 *m/z* window. Each sample was analyzed in 3 biological and 2 technical injection/MS replicates. All MS raw data has been uploaded to the Mass spectrometry Interactive Virtual Environment (MassIVE) site and can be downloaded from the MassIVE website at http://massive.ucsd.edu/ProteoSAFe/datasets.jsp, MassIVE ID# MSV000079335.

### Bioinformatic database searches

All mass spectrometric data was searched using ProteinPilot 4.5 (rev. 1656) with the Paragon algorithm 4.5.0.0, 1654 (SCIEX) [45] using a custom database downloaded from the Kyoto Encyclopedia of Genes and Genomes (KEGG) website (http://www.genome.jp/kegg/) consisting of 1999 protein sequences from *N*. *gonorrhoeae* strain FA 1090 (file dated 2/20/2008). Search parameters included: alkylation with iodoacetamide, trypsin as proteolytic enzyme and the “biological modifications” and “thorough search” features were selected. For acetyl enriched peptide fractions the feature “acetylation emphasis” was chosen as additional parameter. The Proteomics System Performance Evaluation Pipeline (PSPEP) tool was used to generate the FDR analyses using a concatenated forward and reverse decoy database to search the data [45]. For identification of acetylated peptides, we required a peptide confidence cut-off threshold of 99%. For whole lysate analysis (protein level quantitation), only proteins with at least two unique peptides with ≥99% confidence were included in our final datasets. The output files from the Protein Pilot searches can be accessed using the MassIVe site listed above.

### Quantitative Skyline MS1 filtering and data analysis

MS1 chromatogram-based quantitation was performed in Skyline 2.6, an open source software project (http://proteome.gs.washington.edu/software/skyline) [46] using the MS1 ion intensity chromatogram processing algorithm MS1 Filtering [47], as previously described [48]. Briefly, all raw files acquired in data-dependent acquisition mode were directly imported into Skyline 2.6, and MS1 precursor ions were extracted for all peptides present in the comprehensive MS/MS spectral libraries that had been generated from corresponding database searches. Quantitative analysis for acetyl-enriched samples was based on extracted ion chromatograms (XICs) and the resulting precursor ion peak areas for M, M+1, and M+2; final quantitative comparisons were typically based on only the highest ranked (naturally most abundant) precursor ion, which was then compared between different conditions.

A Skyline spectral library file that contains all MS/MS spectra of confidently identified acetylated peptides was transferred to the public data-sharing Panorama site [49]. Panorama provides an interactive web-based spectral viewer of all K-acetyl-containing peptides identified in this study that can be accessed at https://panoramaweb.org/labkey/NGackA.url (for all mass spectrometric identification details also see S2–S4 Tables).

### Statistical analysis of quantitative MS data sets

All peak areas obtained from extracted precursor ion chromatograms using MS1 Filtering were exported from Skyline into Excel spreadsheets using the statistical program ‘MS1Probe’ [50], (http://skytools.maccosslab.org). For each biological replicate and strain, the peak areas for individual K-acetyl peptides were automatically assembled in a data array to subsequently calculate peak area ratios per peptide between the two strains, i.e. 1291*ackA*/1291wt (for each strain, the 2 technical MS replicates were grouped). Significance was assessed using two-tailed Student’s t-test requiring p-values<0.05. To assess possible regulation of a given K-acetyl-containing peptide in the two strains, we set a threshold of at least a two-fold change in the relative peak areas between the *ackA* mutant and the wt strains with a significance of p<0.05 within each biological replicate (each with 2 technical MS replicates). To be considered a “candidate”, such significant upregulation was then required in at least two independent biological replicates out of the three biological replicates. The overall average ratio for each candidate peptide was generated by log-transforming the individual biological replicate ratios, generating an overall ratio, and finally converting back to a natural ratio. In cases where a specific K-acetyl site was identified with different precursor ion charge states or that produced multiple K-acetyl containing peptides with additional missed proteolytic cleavages or modifications, the most reproducible and most robustly ionizing species per site was chosen for quantitation. To determine if the relative abundance of acetylated proteins that showed candidate acetylation sites for acetyl regulation was similar or different in the mutant relative to the parent strain, levels were assessed by quantifying non-modified peptides from the whole cell lysates. The peptides generated from the lysates were assessed using the data-independent SWATH acquisitions in Skyline [23]. At least four product ions per peptide and a minimum of two peptides per protein were necessary for a given protein to be included in the final protein level quantitation.

### Data analyses tools

Pathway analyses were performed using the functional annotation tool available on the DAVID bioinformatics database website (https://david.ncifcrf.gov/summary.jsp) [51, 52].

Consensus sequences and motifs for all AckA-dependent lysine acetylation sites were generated using the mass spectrometric data. In the present study, 109 unique sites were designated as AckA-dependent. Of these, 6 sites were less than 10 residues from the N- or C-terminus and were removed prior to the motif analysis, leaving 103 acetylated sites. Sequences of 21 amino acids, representing 10 amino acids N-terminal and 10 amino acids C-terminal relative to the acetylated lysine were obtained and designated “motifs”. These motifs were analyzed using IceLogo [53] and the *N*. *gonorrhoeae* proteome was used as the reference set.

Comparisons of *E*. *coli* and *N*. *gonorrhoeae* protein sequences, from functionally annotated genes, were performed using the EMBOSS Water protein alignment tool using the default settings (http://www.ebi.ac.uk/Tools/psa/emboss_water/). Additional sequence alignments were performed using the BLAST tool (protein blast) from NCBI (http://blast.ncbi.nlm.nih.gov/) against either the non-redundant protein sequences or the Protein Data Bank (PDB) protein database. Further annotation of proteins listed as hypothetical were performed with the *N*. *gonorrhoeae* strain 1291 database (file dated 03/04/2015) downloaded from the Broad Institute website (*Neisseria gonorrhoeae* Comparative Initiative, Broad Institute, http://www.broadinstitute.org/annotation/genome/neisseria_gonorrhoeae/MultiHome.html) using batched BLAST searches with an expectation value threshold of 1e-5 or less. These BLAST searches gave additional annotations for 36 acetylated proteins (S5 Table), including the heme utilization protein (NGO1318).

### Structural analysis of AckA-dependent acetylation sites

All crystal structures were obtained from the Protein Data Bank (PDB) and were manually inspected and annotated for matching lysine residues using MacPyMOL version 1.7.6.1. Structures from *Neisseria* species were preferentially used, but when not available the proteins with the highest homology and with conserved lysine residues of interest were used.

## Results and discussion

### Western blot

**Fig 2:**
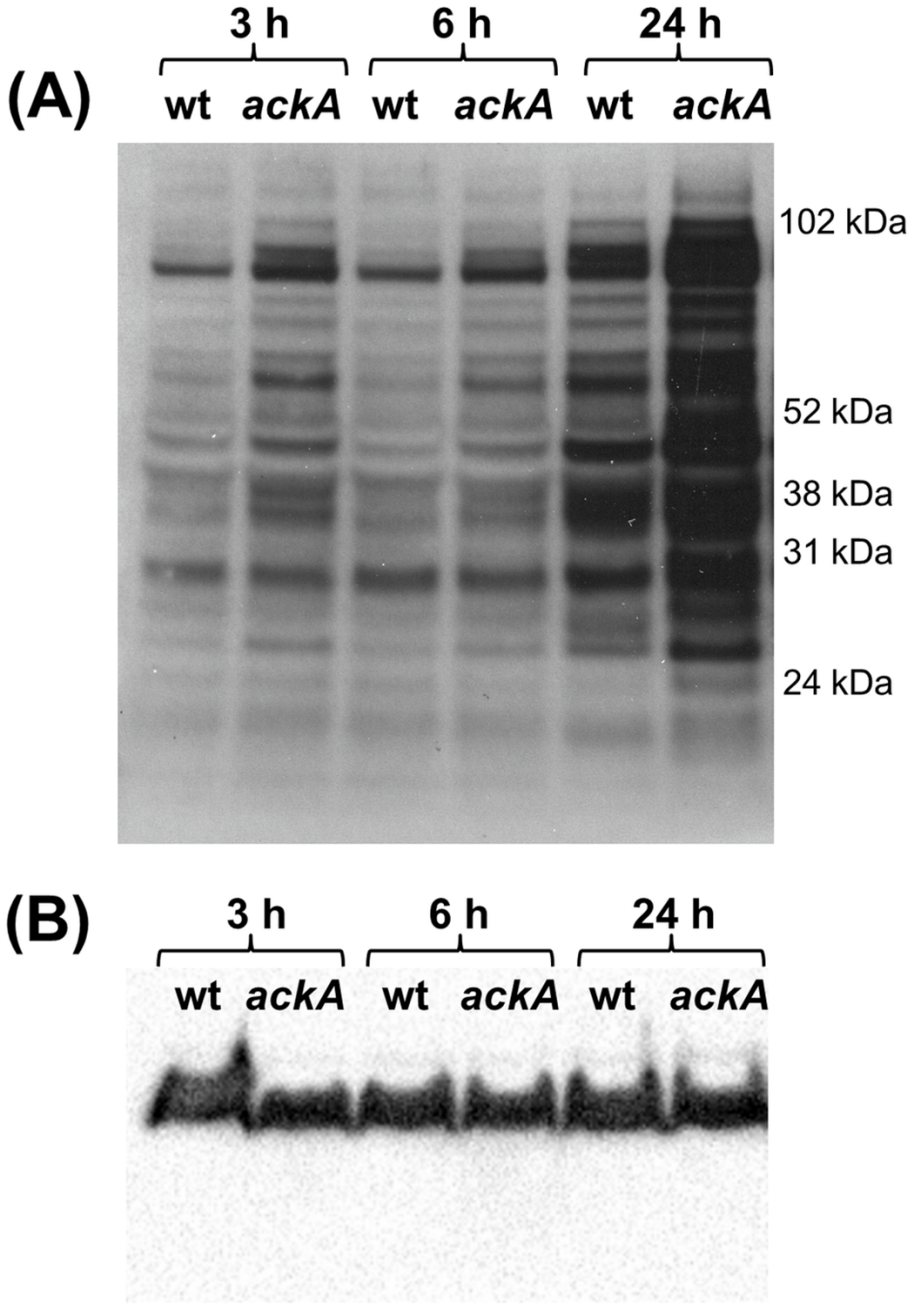
Western blots with (A) MAb Acetyl-K(103) and (B) MAb 2C3 loading control. Samples were taken from broth-grown cultures of 1291wt and 1291*ackA* at 3, 6, and 24h. The MAb Acetyl-K(103) blot shows that acetylation levels in 1291*ackA* are higher at every time-point compared to its parent strain 1291wt. The 2C3 blot confirms that equivalent levels of protein were used in these experiments.

In our initial efforts to determine if lysine acetylation was present in *N*. *gonorrhoeae*, whole cell lysates from 1291wt grown for 3, 6, and 24 h were examined by Western blot using an anti-acetyl-lysine antibody (Fig 2). These analyses demonstrated that lysine acetylation is present in *N*. *gonorrhoeae*. To determine if the levels of AcP impact overall lysine acetylation levels, a 1291*ackA* mutant was generated and its whole cell lysate from 3, 6, and 24 h cultures were examined for lysine acetylation. There was a higher level of acetylation in the *ackA* lysates compared to the wt lysates at all growth time-points (Fig 2A). The highest levels of acetylation for both the wt and *ackA* mutant strains were observed at the 24 h time-point (Fig 2A). The loading control blot confirmed that equivalent protein amounts were loaded for each sample (Fig 2B). These data show that lysine acetylation is present in *N*. *gonorrhoeae* and that at least some of these acetylated sites are impacted by the level of AcP, which is central in non-enzymatic acetylation.

### Mass spectrometric identification of lysine-acetylation sites

**Fig 3:**
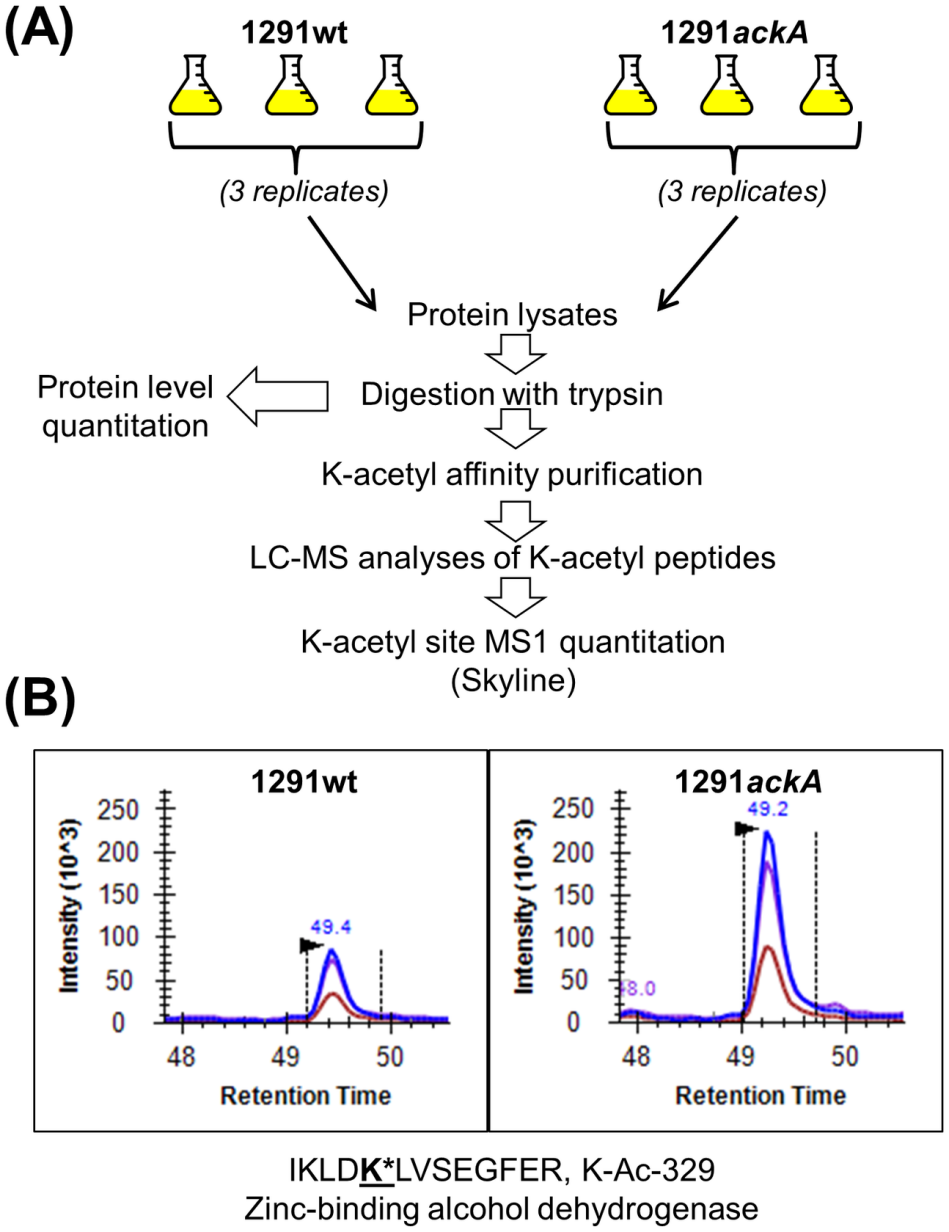
Mass spectrometric workflow. (A) Three biological replicates of *N*. *gonorrhoeae* strains 1291wt and 1291*ackA* were grown overnight in broth cultures. Cells were harvested, washed, and a protein lysate was generated and digested with trypsin. Affinity enrichment for acetyl-lysine (K-acetyl)-containing peptides was performed using a polyclonal anti-acetyl-Lys antibody. Enriched K-acetyl peptides were analyzed by high-resolution label-free LC-MS/MS and quantified using MS1 quantitation. (B) An example of a peptide analyzed by MS1 quantitation using Skyline. MS1 ion chromatograms of the precursor ions for M, M+1, and M+2 for the acetylated peptide IKLDK*LVSEGFER, at K-329, from the zinc-binding alcohol dehydrogenase protein for 1291wt and 1291*ackA* are shown. MS1 quantitation was performed by extracting and measuring the peak areas from the most intense isotopic precursors and subsequently comparing the wt and *ackA* mutant peptides.

**Fig 4:**
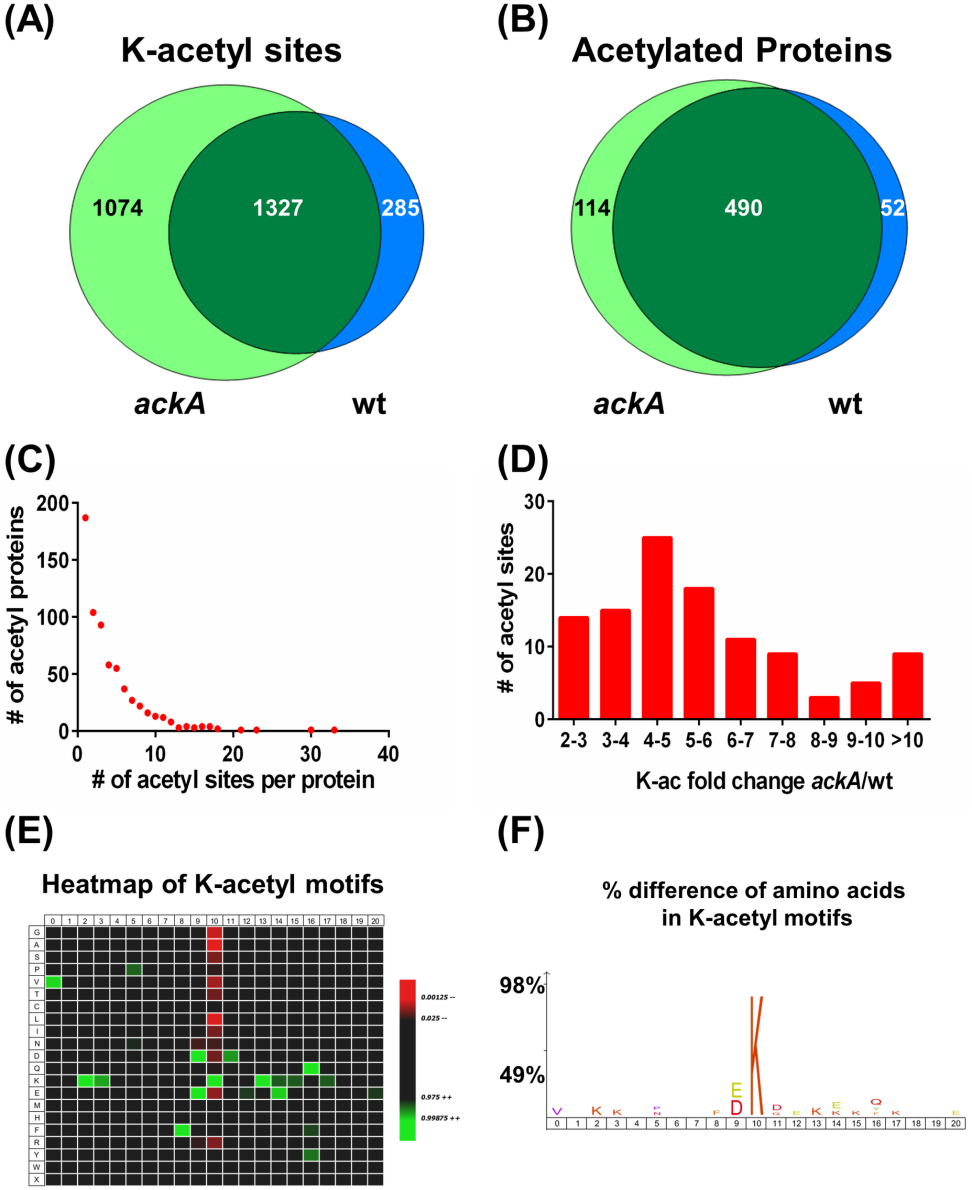
Analysis of acetylation sites detected in the present study. A total of 2686 unique acetylation sites from 656 unique acetylated proteins were observed in the current study. Venn diagrams showing the overlap of 1291wt and 1291*ackA* (A) acetylation sites and (B) acetylated proteins detected in this study. (C) The number of acetylation sites detected per protein was graphed. (D) Lysine acetylation sites that were observed in two or more biological replicates with a 2-fold or greater *ackA*/wt ratio and a p-value of <0.05 were designated as “AckA-dependent”. The fold change distribution for these 109 “AckA-dependent” acetylation sites from 70 proteins was then graphed. (E-F) IceLogo generated outputs showing the analysis from the +/-10 amino acids relative to the acetylation sites from all “AckA-dependent” sites. In the heatmap output (E) the color schema generated shows the p-value score for significant amino acid positions. Amino acids highlighted in red indicate that this position is less likely to occur, and those highlighted in green are 99% more likely to occur. In the percentage difference output (F), the amino acids shown occur in that position with a significantly higher frequency than what would be expected based on the amino acid frequency in the whole proteome reference set. The larger the letter the higher the preference is for a particular position in the AckA-dependent motifs.

In an effort to identify sites of acetylation in *N*. *gonorrhoeae*, broth cultures of 1291wt and 1291*ackA* were grown in triplicate (Fig 3A). The cell pellets were lysed, and peptides were generated by proteolytic digestion with trypsin. Then affinity enrichment, using a polyclonal anti-acetyl-lysine antibody, was performed on 1 mg of each sample (Fig 3A). The acetyl-enriched samples were analyzed, using two injection replicates, on an orthogonal time-of-flight mass spectrometer, the TripleTOF 5600. Data-dependent acquisitions were searched against a custom *N*. *gonorrhoeae* strain FA 1090 database using Protein Pilot (S2 Table). Overall we identified 2686 total unique acetylation sites from 656 unique proteins (S3 and S4 Tables). These numbers are comparable to a similar study done by our group with *E*. *coli* where a total of 2730 unique acetylation sites from 806 proteins were detected [23]. In the 1291wt samples, we identified 1612 unique acetylation sites from 542 unique proteins (S3 and S4 Tables), while for the *ackA* samples, we found 2401 unique acetylation sites from 604 unique proteins (S3 and S4 Tables), representing a 1.5-fold increase in the number of acetylation sites in the 1291*ackA*/1291wt samples. There were a total of 1327 sites in common, with 1074 sites detected only in the *ackA* samples and 285 sites found exclusively in the wt samples (Fig 4A, S3 Table). A total of 490 acetylated proteins were found in common, with 114 acetylated proteins seen only in the *ackA* samples, and 52 acetylated proteins observed in only the wt samples (Fig 4B, S4 Table).

In the current *N*. *gonorrhoeae* study, as well as two previous *E*. *coli* studies [23, 31], a higher number of acetylated peptides were identified in the *ackA* mutants compared to their wt counterparts. The acetate kinase (AckA) is part of the AckA-Pta pathway, which reversibly converts acetate and AcCoA to yield the high-energy intermediate AcP (Fig 1). In addition to analyzing the *ackA* mutants, the previous *E*. *coli* studies also examined mutants in *pta* to more clearly define which components of the pathway were involved in increasing acetylation levels [23, 31]. Weinert *et al*. demonstrated that the acetylation levels of exponentially grown cells or growth-arrested cells from a *pta* mutant were substantially reduced compared to their wt counterparts [31]. In a related study, Kuhn *et al*. demonstrated that exposure of a *pta* mutant to exogenous acetate in the growth medium led to an increase in acetylation levels compared to the *pta* mutant grown without exogenous acetate [23]. The *E*. *coli* studies also demonstrated that cells unable to convert AcP to acetate, such as the *ackA* mutant, showed increased acetylation levels compared to their wt counterparts. These data combined with the acetate-dependent acetylation in the *pta* mutant demonstrated that the level of AcP affects acetylation. Since an *ackA* mutant increases the level of AcP, it seems logical that the increase in protein acetylation seen in the *N*. *gonorrhoeae ackA* mutant is due to an increased level of available AcP. Attempts by our group to generate a *pta ackA* double mutant in *N*. *gonorrohoeae* were unsuccessful; therefore, knocking-out both of these genes appears to be lethal in the gonococcus.

Distribution of the acetylation sites was not uniform among all the proteins (Fig 4C). Most of the acetylated proteins identified in this study had multiple acetylation sites, with 72% of the acetylated proteins having >1 acetylation site. This observation is similar to what our group observed in previous *E*. *coli* and mitochondria studies and suggests that acetylation may have multiple sites for regulation [23, 48, 54]. Four proteins had more than 20 acetylation sites, including the chaperonin 60 kDa subunit GroEL protein and the chaperone protein DnaK, with 30 and 33 acetylation sites each, respectively (Fig 4C, S4 Table). GroEL has been previously identified in a number of bacteria as one of the most highly acetylated proteins; however, the reason for this high level of acetylation remains unclear [36]. Additionally, in a recent review, it was reported that nearly all of the published bacterial acetylomes showed that GroEL, elongation factor G (EF-G), and enolase were acetylated [36]. Acetylation sites were found in all three of these proteins in the current study, further supporting the idea that acetylation plays an important role in these proteins.

### Label-free quantitation of lysine acetylation sites

MS1 Filtering was used to quantitatively compare the relative abundances of the lysine-acetylation sites in wt versus *ackA* samples (Fig 3B). A pairwise comparison of the peak areas for each set of three biological replicates, with two technical injection replicates each, was then performed (Fig 3B). If the ratio between the *ackA*/wt was >2, at a p-value <0.05, and was found in two or more biological replicates, it was designated as “AckA-dependent” and an average ratio was generated for each site. A total of 109 unique AckA-dependent acetylation sites from 70 unique proteins were detected in this study (S6 Table). Of these sites, 14 met the above AckA-dependent threshold criteria, in all three biological replicates, and 91 sites met the threshold criteria in two biological replicates. The remaining four sites were observed in all three replicates as having an increase in acetylation in 1291*ackA* compared to 1291wt, but only two of the four replicates met the 2-fold cut-off threshold. None of the AckA-dependent sites had acetylation ratio data that was contradictory to an increase in acetylation of the *ackA* mutant compared to the wt strain. The reason for the acetylated sites not being observed and quantified in all three biological replicates is likely due to detection, it is essentially a dynamic range issue. We implemented our high threshold standards for the AckA-dependent acetylation sites to ensure a high level of confidence for these sites.

Of proteins with AckA-dependent acetylation sites, 25 had more than one AckA-dependent acetylation site (S6 Table). The average *ackA*/wt ratios for lysine acetylation sites ranged from 2.3–16.8 and approximately half of the sites showed ratios that were greater than 5 (Fig 4D). The ribosome recycling factor protein (Rrf), the regulator of *pilE* expression protein (RegF), the phosphoglycerate mutase protein (PgmA), and the GroES chaperonin 10 kDa subunit protein all had at least one acetylation site with a K-acetyl *ackA*/wt ratio >10 (S6 Table). Parallel experiments of the whole cell lysates from *ackA* and wt were used to examine potential protein level changes. We did not observe any significant change in the protein levels of the proteins that contained AckA-dependent acetylation sites that could account for the differences seen in the acetylation levels.

### Pathway analyses of acetylated proteins

Functional analyses of all proteins with AckA-dependent acetylation sites were performed using the DAVID functional analysis tool. GO terms, keyword, and KEGG pathway analyses were performed (S7–S9 Tables). The keyword analyses demonstrated a significant enrichment for a number of categories including: protein synthesis, ribosomal proteins, RNA binding, and rRNA binding (S7 Table). Similarly, GO term analyses showed a significant enrichment for the following terms: translation, ribosome, ribonucleoprotein complex, and ribosomal structural proteins (S8 Table). The GO term analyses also found that the term “structural molecule activity” was significantly enriched in the proteins with AckA-dependent acetylation sites (S8 Table). KEGG pathway analyses showed enrichment for glycolysis, ribosomes, and to a lesser extent, the TCA cycle (S9 Table). Enrichment for ribosome, glycolysis, and TCA cycle proteins has also been observed in an *E*. *coli ackA* mutant [23] and several other bacterial studies [22, 24, 30–33, 48, 55]. One proposed explanation for the high levels of acetylation amongst central metabolism proteins is that acetylation of key enzymes involved in these pathways may allow cells to cope with varying nutrient states [23, 48].

### Conservation of AckA-dependent acetylation sites across species

To determine if AckA-dependent acetylation sites that were found in *N*. *gonorrhoeae* were conserved across species, we compared them with AckA-dependent acetylation sites previously found in *E*. *coli* [23]. Matches between the *N*. *gonorrhoeae* and *E*. *coli* proteins were based on functional annotations of their corresponding genes and on protein sequence alignments. The alignment of the protein sequences was also used to determine common acetylation sites. Sixteen AckA-dependent acetylation sites were found in common between *N*. *gonorrhoeae* and *E*. *coli* (S10 Table); this represents 15% of the total AckA-dependent K-acetyl sites identified in this study. Of these, the phosphoglycerate mutase protein (PgmA), the ribosome recycling factor protein (Rrf), and the phosphoglycerate kinase protein (Pgk) had two or more AckA-dependent sites in common. The conservation of these sites across bacterial species suggests that they may be important regulation points for these proteins.

### Motif analyses

The amino acid sequences +/-10 positions from AckA-dependent lysine-acetylation sites were examined using IceLogo and a heatmap and percentage logo were generated. Sequences less than 10 amino acids from the N- or C-terminus of a protein were not included in the analyses. Motifs were generated from a total of 103 sequences for all AckA-dependent acetylation sites. In the heatmap output (Fig 4E), the color schema generated shows the p-value score for significant amino acid positions. Amino acids highlighted in red indicate that this position is less likely to occur, and those highlighted in green are 99% more likely to occur. Similarly, the differences between the frequency of a particular amino acid in the AckA-dependent acetylation site set and *N*. *gonorrhoeae* whole proteome reference set are shown in the percentage logo. The amino acids shown in Fig 4F are ones that occur with a significantly higher frequency than what would be expected based on the *N*. *gonorrhoeae* whole proteome reference set. The larger the letter in the motif the higher the preference is for that amino acid to occur in the designated position in the AckA-dependent motifs. The motif generated from the AckA-dependent lysine acetylation sites showed a high prevalence for the negatively charged amino acids aspartate (D) and glutamate (E) at the -1 position and to a lesser degree, aspartate (D) at the +1 position. A similar preference for aspartate and glutamate at the -1 position relative to AckA-dependent K-acetyl sites was previously observed in *E*. *coli* [23].

### Growth comparisons

**Fig 5:**
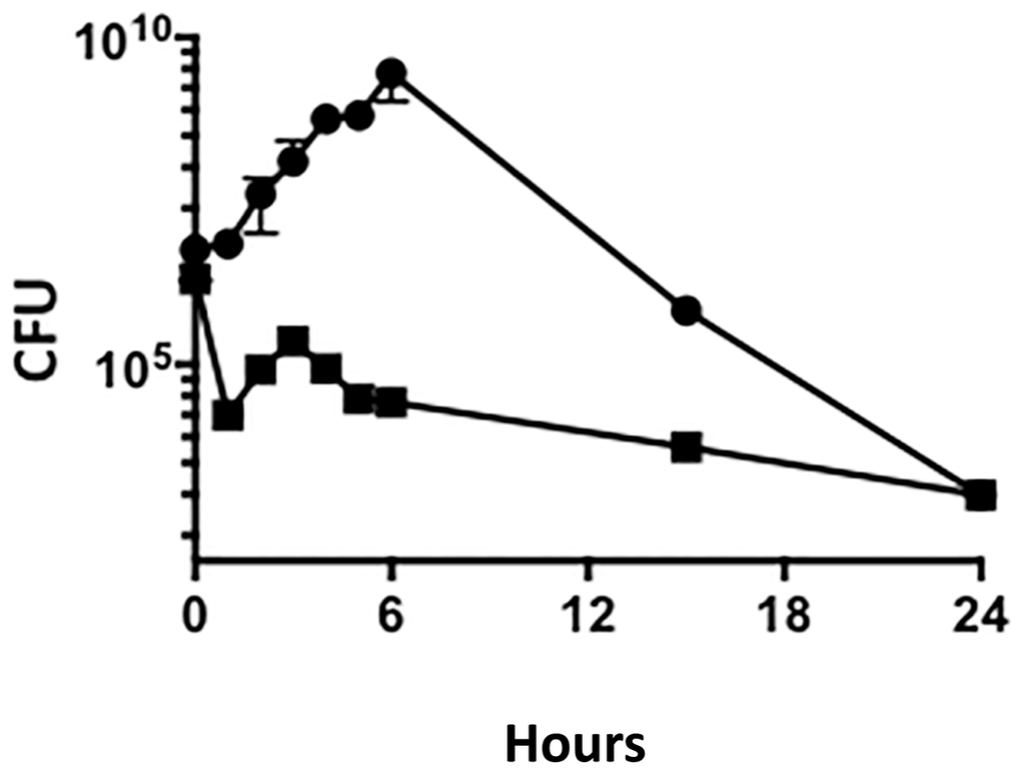
Growth comparisons of 1291wt (■) and 1291*ackA* (●) from aerobically grown broth cultures. Samples were taken at 0, 1, 2, 3, 4, 5, 15, and 24 h, plated in triplicate, and the average colony forming units (CFU) determined for each time-point. Duplicate experiments were performed and the results presented are the average of those two values. As can be seen, aerobic growth of 1291*ackA* is limited in liquid media compared to its parent strain 1291wt.

To assess the impact of the *ackA* mutation on bacterial growth under aerobic conditions, standard 24 h growth curves of both 1291wt and 1291*ackA* were performed and the CFUs determined at time-points throughout the growth curve (Fig 5). The CFU data from the wt and *ackA* strains shows that there appears to be an overall growth defect in the *ackA* mutant since the CFU counts never reach the level of the wt culture.

Previous work showed that anaerobic growth in *N*. *gonorrhoeae* is coupled to nitrite reduction [56]; therefore, anaerobic growth of the gonococcus may be facilitated by the addition of a nitrite to solid media [40]. In the current study, growth of 1291wt and 1291*ackA* strains under anaerobic conditions was performed using GC agar plates containing sodium nitrite. Triplicate plates were inoculated with 5×10^6^ CFU bacteria, followed by incubation for 48 h in an anaerobic chamber. As a control, identical plates of the 1291wt and 1291*ackA* cultures were also grown aerobically. After the 48 h incubation period the growth of the anaerobic and aerobic cultures were assessed. The CFUs of the 1291wt plates on both the aerobically and anaerobically grown plates were too numerous to count. The 1291*ackA* plates grown anaerobically had no CFUs on them; while the 1291*ackA* plate that was grown aerobically had too many colonies to count. These data confirmed that the 1291*ackA* mutant is unable to grow anaerobically. In an effort to more clearly understand which proteins may be important for growth under anaerobic conditions, we compared the proteins found in our study that have AckA-dependent acetylation sites with previous studies where *N*. *gonorrhoeae* was grown under anaerobic conditions (S11 Table) [40, 57, 58]. Comparisons between the current study and the study by Zielke *et al*. found 13 proteins in common between them including the two-component response regulator, MisR, the putative regulator of *pilE* expression, RegF, and a number of proteins involved in metabolic pathways (S11 Table). This list of proteins represents a group of proteins that appear to be impacted by changes in metabolism and growth conditions.

**Fig 6:**
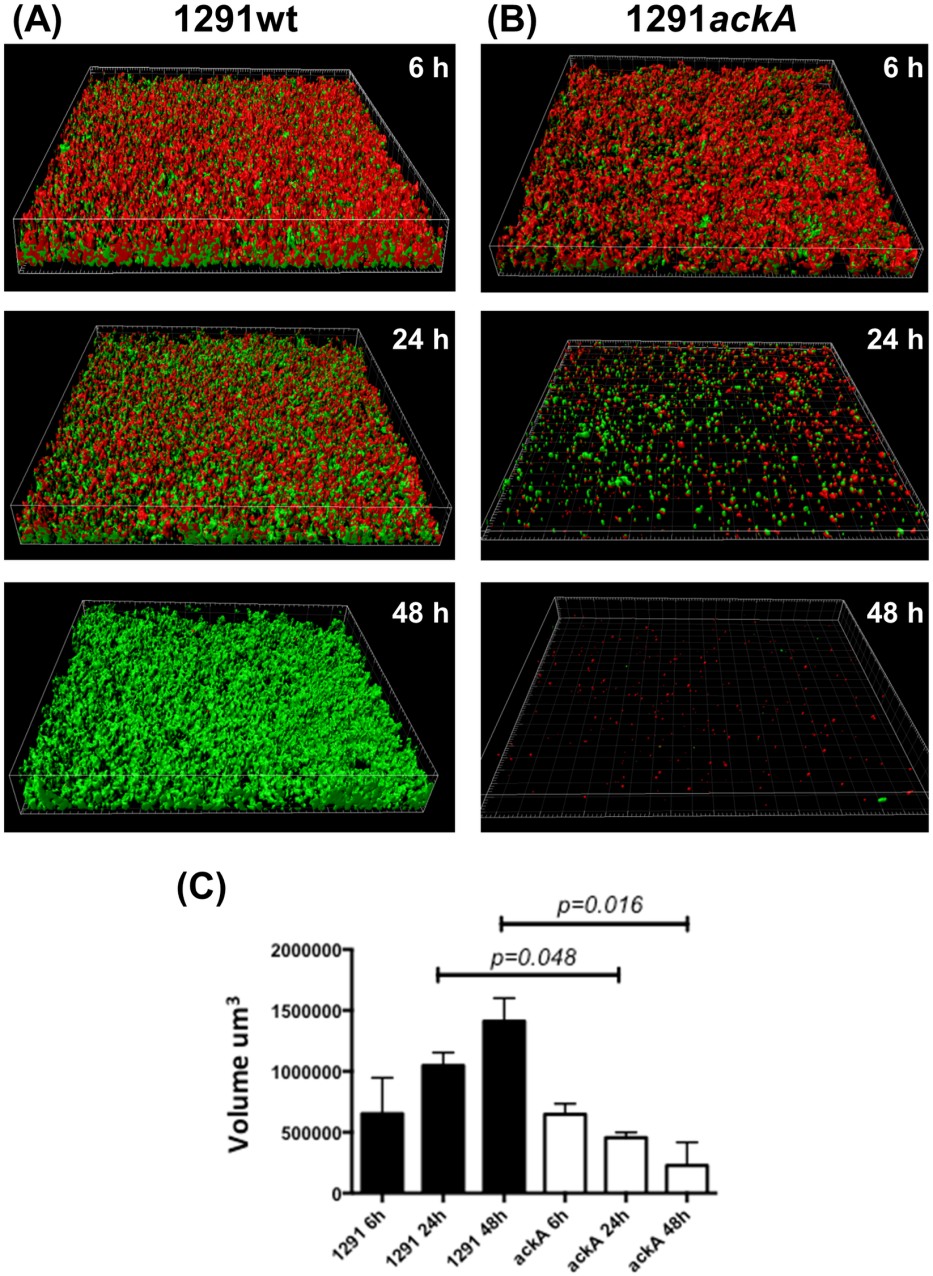
Biofilm growth comparison. Stacked z-series of biofilms from various time-points stained with LIVE/DEAD stain (green/red). (A) 1291wt biofilms grown for 6, 24, or 48 h. (B) 1291*ackA* biofilms grown for 6, 24, or 48 h. These images show that 1291wt is able to form a confluent biofilm that remains viable at 48 h. In contrast, the 1291*ackA* mutant is initially able to form a biofilm, but by 24 h it is no longer viable. (C) The volume of the biofilms were also determined and analyzed using a Mann-Whitney comparison. A significant difference between overall size of the 1291wt and 1291*ackA* biofilms were seen at 24 h and 48 h (with p-values of 0.048 and 0.016, respectively). Biofilm experiments were performed in duplicate in two independent experiments.

To assess the impact of the *ackA* mutation on biofilm formation and maturation, biofilms of 1291wt and its corresponding *ackA* mutant were grown for 6, 24, and 48 h. The biofilms were subsequently stained with LIVE/DEAD stain, which stains viable cells green and dead cells red, and then examined by confocal microscopy. The micrographs from these experiments are shown in Fig 6. These images clearly show a confluent layer of the wt cells at all time-points. Images of the wt 6 and 24 h time-points (Fig 6A) show that a percentage of the population is dead (red). The wt image from the 48 h time-point (Fig 6A) shows that the cells are uniformly living (green). All three 1291wt time-points (Fig 6A) show that the number of living cells (green) increases over time. Similar to the wt 6 h biofilm, the 6 h *ackA* mutant biofilm (Fig 6B) grew to confluence and contained a mixture of alive (green) and dead (red) cells. In contrast to the wt 24 h biofilm, the 24 h *ackA* mutant biofilm (Fig 6B) was almost completely absent, indicating that this mutant was unable to maintain a biofilm. This was further illustrated in the image from the 48 h time-point from the *ackA* mutant where a biofilm was no longer present (Fig 6B). These data indicate that the *ackA* mutant can initiate, but not sustain a viable biofilm. The overall mass and height of the 1291wt and 1291*ackA* biofilms were also compared using data from stacked microscopy images (Fig 6C). Statistical comparison of these volumetric measurements showed that there was a significant difference in the overall size of the wt and *ackA* biofilms at 48 h (p-value = 0.016) and to a lesser extent at 24 h (p-value = 0.048). These measurements offer further support that the 1291*ackA* biofilm is able to form initially, but it does not remain viable over time.

The effects of an *ackA* mutation on biofilm formation have also been examined in *E*. *coli* [59, 60]. These experiments demonstrated that the *ackA* biofilm biomass was influenced by temperature, time, and carbon source [59]. Relative comparisons of the biofilm biomasses of the *E*. *coli ackA* mutant with its wt counterpart demonstrated that the *ackA* mutant had a similar, larger, or smaller relative biomass depending on the growth conditions [59, 60]. It has been proposed that biofilm formation may be affected by components of the central metabolism pathway modifying signal transduction pathways such as two-component response regulators in response to environmental conditions [61]. Until further studies are done, it is unclear if the various growth deficiencies seen in the *N*. *gonorrhoeae ackA* mutant are due to metabolic defects caused by the mutation, to changes in the degree of acetylation of certain proteins, or due to a combination of these and other factors.

### Mapping of detected acetylation sites onto crystal structures

**Fig 7:**
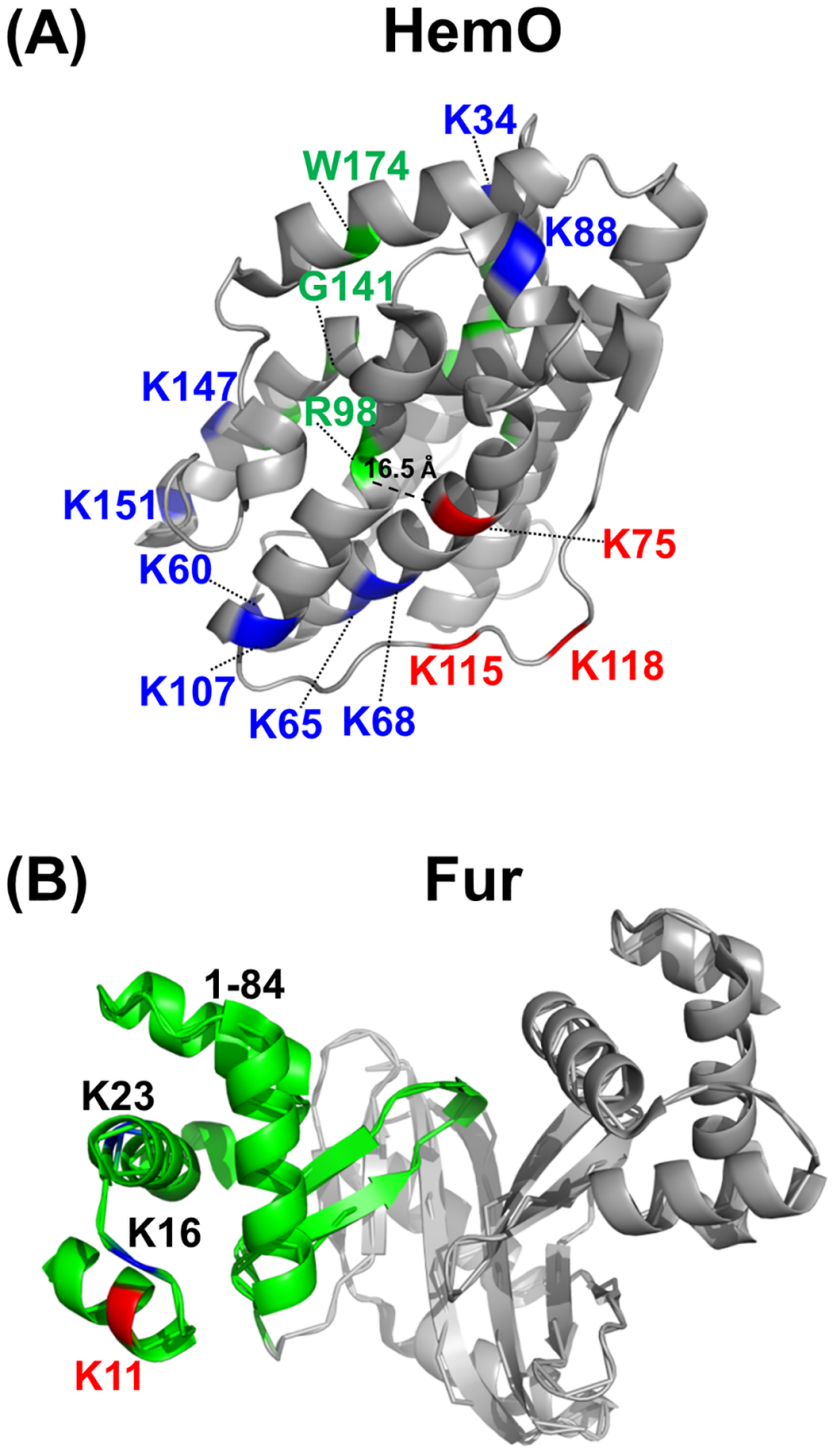
Detected acetylation sites were mapped onto the crystal structures that had the highest homology to the gonococcal iron binding and iron regulation proteins HemO and Fur. (A) Crystal structure of HemO from *N*. *meningitidis* (PDB: 1P3T). Possible heme binding sites and the putative O_2_ activation site (R98) are shown in green. The distance between the predicted O_2_ activation site (R98) and the closest AckA-dependent K-acetyl site (K75) was measured to be 16.5 Å. (B) Crystal structure of Fur from *V*. *cholera* (PDB: 2W57). A proposed DNA-binding site (residues 1–84) is shown in green. All three of the common K-acetyl sites mapped to the predicted DNA-binding region of Fur. In both panels K-acetyl sites shown in red were found to be AckA-dependent, and all K-acetyl sites shown in blue were detected in the current study. All residue positions of common K sites are listed as their position in their respective *N*. *gonorrhoeae* proteins. Protein databank (PDB) identifications are listed for each structure.

*N*. *gonorrhoeae* is a strict human pathogen. Some of the AckA-dependent K-acetyl sites observed in this study were found in proteins associated with gonococcal pathogenesis. Iron acquisition and uptake is critical to the survival and propagation of *N*. *gonorrhoeae* within the host [62]. Pathogenic *Neisseria* have a number of receptors for iron acquisition and possess mechanisms for tightly regulating intracellular iron levels [62]. The crystal structures of two proteins involved in iron acquisition were retrieved and the observed K-acetyl sites were mapped onto the structures (Fig 7). One of these proteins, NGO1318, a predicted heme utilization/heme oxygenase protein (HemO, S5 Table), was identified with eleven K-acetyl sites, three of which were designated as AckA-dependent. Two studies by Zhu *et al*. demonstrated that HemO was necessary for both the utilization and degradation of heme in *Neisseria* [63, 64]. The K-acetyl sites found in the current study, as well as the predicted binding/activation sites [65, 66] were mapped on the previously determined *N*. *meningitidis* HemO crystal structure (Fig 7A) [65]. The AckA-dependent K-acetyl site at K-75 was the closest AckA-dependent K-acetyl site to the predicted O_2_ activation site at Arg-98 [65] with a calculated distance between the sites of 16.5 Å. We also identified seven K-acetyl sites, including two AckA-dependent sites, in the ferric uptake regulator (Fur) protein. After sequence comparisons between the Fur proteins of *N*. *gonorrhoeae* and *Vibrio cholera*, three K-acetyl sites, including one AckA-dependent site, were found in common. These sites, as well as a DNA-binding region, were mapped onto the *V*. *cholera* Fur crystal structure (Fig 7B) [67]. All of the common K-acetyl sites mapped to the predicted DNA binding region of Fur. The conservation of these sites and their location in a region of interest make them potentially interesting targets for future experiments.

## Conclusions

In summary, this study demonstrates that *N*. *gonorrhoeae* extensively acetylates a number of proteins involved in various cellular processes. Similar to previous studies, we found that amongst proteins with AckA-dependent sites there was enrichment for ribosome, glycolysis, and TCA cycle proteins. Additionally, AckA-dependent sites were also found in a number of proteins potentially involved in gonococcal pathogenesis. These proteins included the two-component response regulator, MisR, three pilus associated proteins: RegF, PilT, and PilZ, and two iron acquisition proteins: Fur and HemO. These data present a valuable resource; they provide a number of potential targets for future experiments that will enable us to gain a better understanding of the role of acetylation in pathogenesis and biofilm formation.

## Supporting information

S1 Table Primers used in this study. (DOCX)

S2 Table Mass spectrometric details for all identified acetyllysine-containing peptides identified with at least a confidence score of 99. (XLSX)

S3 Table All lysine acetylation sites and their corresponding motifs for all unique sites detected in all replicates. (XLSX)

S4 Table Acetylated proteins and number of acetylated sites detected in each protein overall. (XLSX)

S5 Table Comparisons of protein annotations from strains FA 1090 and 1291 of hypothetical proteins identified in FA 1090. (XLSX)

S6 Table Average ratios of regulated acetylation sites. Regulated sites were defined as ones seen in at least two biological replicates, with at least a 2-fold increase in the acetylation level and a p-value< 0.05.
(XLSX)

S7 Table Keyword enrichment for acetylated proteins with regulated acetylation sites. (XLSX)

S8 Table GO term enrichment for acetylated proteins with regulated acetylation sites. (XLSX)

S9 Table Kegg pathway enrichment for acetylated proteins with regulated acetylation sites. (XLSX)

S10 Table Regulated lysine acetylated sites that are conserved *in N*. *gonorrhoeae* and *E*. *coli*. (XLSX)

S11 Table (Sheet A) List of proteins found in common between the AckA-dependent K-ac sites found in the current study with a previous study examining protein level changes between anaerobic/aerobic growth. A number of proteins were found in common between these studies. These proteins may represent proteins that are very responsive to changes in metabolism and growth conditions. (Sheet B) List of proteins found in common between the AckA-dependent K-ac sites found in the current study with two previous studies examining: (1)Protein level changes between biofilm/planktonic growth of *N*. *gonorrhoeae* and (2) RNA-seq data comparing gonococcal mRNA levels from bacteria grown anaerobically/aerobically. It was difficult to draw much correlation between these datasets and the current study. The proteins from the biofilm dataset that matched our proteins list had differences that were highly variable or lacked statistical significance. In addition, mRNA levels are known to traditionally correlate poorly with protein-level changes.
(XLSX)
