# Supplementary material for: Identification and characterization of AckA-dependent protein acetylation in Neisseria gonorrhoeae
Source: PLoS One. 2017 Jun 27;12(6):e0179621. doi: 10.1371/journal.pone.0179621 (PMC5487020; doi:10.1371/journal.pone.0179621)
Supplement: S1 Table — (DOCX) [file pone.0179621.s001.docx]

**S1 Table. Primers used in this study.**

| **Name** | ***^a^*Primer Sequence (5’-3’)** | **Description** |
| --- | --- | --- |
| JRH319 | CATCGTTTTCCTTCTCTTTTTCA | NGO0977 upstream forward |
| JRH324 | AAACCTGTCGTTTTGGATTTTT | NGO0977 downstream reverse |
| JRH323 | tgctgaagatcagttgggtgTAAGGTTTTATCCTCACGCGAAC | NGO0977 downstream forward-KAN overhang |
| JRH320 | tacctgctttctctttgcgcCATTGGAATCGGACACGGATA | NGO0977 upstream reverse-KAN overhang |
| JRH321 | tatccgtgtccgattccaatgGCGCAAAGAGAAAGCAGGTA | KAN forward-0977 upstream overhang |
| JRH322 | gttcgcgtgaggataaaccttaCACCCAACTGATCTTCAGCA | KAN reverse-0977 downstream overhang |

*^a^*Lowercase sequences in the primers represent the region complementary to the adjacent primer for PCR SOEing.
